# The detrimental effects of heavy metals on tributaries exert pressure on water quality, *Crossocheilus aplocheilus*, and the well-being of human health

**DOI:** 10.1038/s41598-024-53340-5

**Published:** 2024-02-04

**Authors:** Muhammad Subhanullah, Nazim Hassan, Sajid Ali, Ibrahim A. Saleh, Muhammad Ilyas, Bakht Rawan, Waheed Ullah, Babar Iqbal, Mohammad K. Okla, Ibrahim A. Alaraidh, Shah Fahad

**Affiliations:** 1 Department of Environmental Sciences, Abdul Wali Khan University Mardan, Mardan, 23200 Khyber Pakhtunkhawa Pakistan; 2 Department of Environmental Sciences, Shaheed Benazir Bhutto University Sheringal, Dir Upper, Khyber Pakhtunkhwa Pakistan; 3 Institute of Applied Ecology, Chinese Academy of Sciences, Shenyang, 028118 Liaoning People’s Republic of China; 4 Government Degree College Gulabad, District Lower Dir, Khyber Pakhtunkhwa Pakistan; 5 Faculty of Science, Zarqa University, Zarqa, 13110 Jordan; 6 International Islamic University, Islamabad, 44000 Pakistan; 7 COMSATS University Islamabad, Abbottabad Campus, Abbottabad, 22060 Pakistan; 8 Institute of Environmental Health and Ecological Security, School of Environment and Safety Engineering, Jiangsu Province Engineering Research Center of Green Technology and Contingency Management for Emerging Pollutants, Jiangsu University, Zhenjiang, 212013 People’s Republic of China; 9 Botany and Microbiology Department, College of Science, King Saud University, P.O. Box 2455, 11451 Riyadh, Saudi Arabia; 10 Department of Agronomy, Abdul Wali Khan University Mardan, Mardan, 23200 Khyber Pakhtunkhwa Pakistan

**Keywords:** Ecology, Environmental sciences

## Abstract

The escalating presence of heavy metals (HMs) in the Panjkora River water and their impact on fish pose a significant challenge to both the ecological community and human health. Consequently, a study was conducted with the primary aim of elucidating their influence on human health-related issues. To address this, the concentrations of heavy metals, including arsenic (As), cadmium (Cd), iron (Fe), manganese (Mn), lead (Pb), and zinc (Zn), in both water and the fish species *Crossocheilus diplocheilus* were investigated across various locations within the study area. The quantification of HMs concentration was carried out utilizing an atomic absorption spectrophotometer. The highest concentration in water was found as 0.060 mg/L for Pb and lowest for Fe, whereas the highest concentration in fish was 2.028 mg/kg for Pb and lowest for As. Human health risk associated with fish eating was evaluated by using health risk indices (HRI) for non-carcinogenic health risks and targeted cancer risk (TR) for carcinogenic health risks. The values of the health risk index (HRI) were found greater than 1 except Fe (0.0792), Zn (0.782), and Mn (0.541). The highest mean HRI > 1 was recorded for As (62.99), Cd (26.85), and Pb (10.56). This implies that fish consumption from river Panjkora is not safe up to some extent. Similarly, the TR value for As, Cd, and Pb was found 2.8 $\times {10}^{-2}$, 1.6 $\times {10}^{-2}$, 2.8 ×${10}^{-3}$ which showed cancer risk. There is a detected risk to human health associated with the consumption of fish from the Panjkora River. The government must implement adaptive measures to address this significant issue of water pollution in the study area. Additionally, there is a need for further extensive and prolonged research studies in this context.

## Introduction

Heavy metals (HMs) are defined by having an elemental density (ED) exceeding 5 g cm^3^ and an atomic number (Z) surpassing 20, as stipulated by Aliand and Khan in 2018. These metals, acknowledged for their non-biodegradable, cytotoxic, mutagenic, or carcinogenic properties, pose significant environmental contamination risks^1–3^. The growth of manufacturing and agricultural industries has notably contributed to the contamination of water with metals, impacting both the environment and living organisms^4,5^. Previous researchers demonstrated that various sources emit heavy metals by contaminating both freshwater and saltwater, thereby affecting aquatic life^6–8^.

Different metals that infiltrate ground and surface water exhibit detrimental effects on aquatic life and human health. For instance, lead (Pb), widely utilized in paints, explosives, protective coatings, building materials, and wine due to its density, malleability, and erosion resistance, is particularly concerning^9^. The pollution of river networks with heavy metals stands out as a major contemporary environmental challenge globally, resulting from a combination of socioeconomic and industrial activities^10,11^. In the context of fluvial environments, this pollution can arise from geological weathering, atmospheric deposition, or the release of industrial, domestic, urban, or agricultural discharges^12,13^. In three-quarters of urban areas in Asia, Africa, and Latin America, industrial or urban wastewater irrigation is a common reality^14^. In today's modern world, environmental contamination is the biggest problem. Among other environmental pollutants, HMs are well-known and cause more worry because of their toxicity to aquatic and terrestrial life^15^. Human activities have released a significant number of HMs into the environment^16–19^. However, HMs, are persistent and harm the health of living things^20,21^. HMs from mining operations agriculture, and industries discharged into rivers are trapped by sediments that transit and bio-magnify HMs causing severe diseases in fish and human beings^22–26^. The heavy metal poisoning of many inland water environments has grown recently, according to Otchere^25^. Even though 70% of the Earth's surface is made up of water, freshwater resources are quickly running out^26^. Further reducing the availability of clean, fresh water sources is the rapid industrialization and lifestyle changes that have led to the contamination of these watery resources with a wide range of pollutants^27^.

Drinking water contaminated with metals can have long-lasting and short-term impacts, including lowered immunity, oxidative stress, gastrointestinal ulcers, and even cancer. Mercury (Hg), Copper (Cu), Zinc (Zn), Arsenic (As), Chromium (Cr), Nickel (Ni), Cobalt (Co), Cadmium (Cd), Lead (Pb), and others are toxic HMs that can be dangerous^28^. Natural and manmade activities, HMs accumulation in fish increase throughout the world, and its potential threat to health^29,30^. Infected drinking water causes over 0.84 million people to die from diarrhea each year, according to a WHO report from 2018. Pakistan, the world's sixth most populous nation, is likewise grappling with severe water^31^. While some of the HMs, such as Pb, Hg, and Cd, are biologically nonessential and highly poisonous to living things, low quantities of others are necessary for the growth and development of living things. Even necessary metals can become harmful if their concentration exceeds the allowable limit^32–34^. Similar to this, metals in sediments can come from a variety of sources, including air deposition, mining deposits, bedrock weathering and erosion, and industrial and agricultural effluents^35,36^. Contaminants that contain HMs constantly enter rivers from rapid urbanization, agricultural activities, and industrialization^37^.

Environmental pollution stands as the primary threat to public health in Pakistan, as highlighted by Shakir^38^. The water bodies within the country frequently exhibit the presence of heavy metals (HMs) such as mercury (Hg), iron (Fe), copper (Cu), zinc (Zn), lead (Pb), cadmium (Cd), nickel (Ni), and arsenic (As). These metals have detrimental effects on aquatic life and become incorporated into the food chain, as indicated by Hussain^39^. While certain heavy metals like lead (Pb), cadmium (Cd), and mercury (Hg) lack known biological functions in fish, others such as iron, zinc, and copper play essential roles in fish metabolism^40^. The primary sources of metals and metalloids, whether natural or anthropogenic, consistently introduce these substances into aquatic bodies, posing significant threats to human and ecological health due to their toxic nature^41^. The release of heavy metals from sediments into water and their accumulation in fish depend on factors such as the solubility of the metals and processes like adsorption or precipitation^42^. The escalating population ratio contributes to the increasing levels of heavy metals, exacerbated by mismanagement of solid waste, agricultural practices, mining activities, and sanitation issues in the study area, negatively impacting the quality of the Panjkora River. The current study focuses on assessing the concentration of heavy metals in water and fish samples from the Panjkora River, aiming to determine the potential health risks associated with heavy metal ingestion through fish consumption in the study area.

## Materials and methods

### Description of study area

The study was carried out in River Panjkora, located in District Dir lower, which lies in the Hindukush range at 71°, 31°–72°, 14′ east and 34°, 37°–35°, 07′ north^43^. It is around 2700 feet above sea level. It is considered a major lifeline of the area, originates from Kohistan in the district of Upper Dir, and flows southward to Lower Dir.

**Figure 1:**
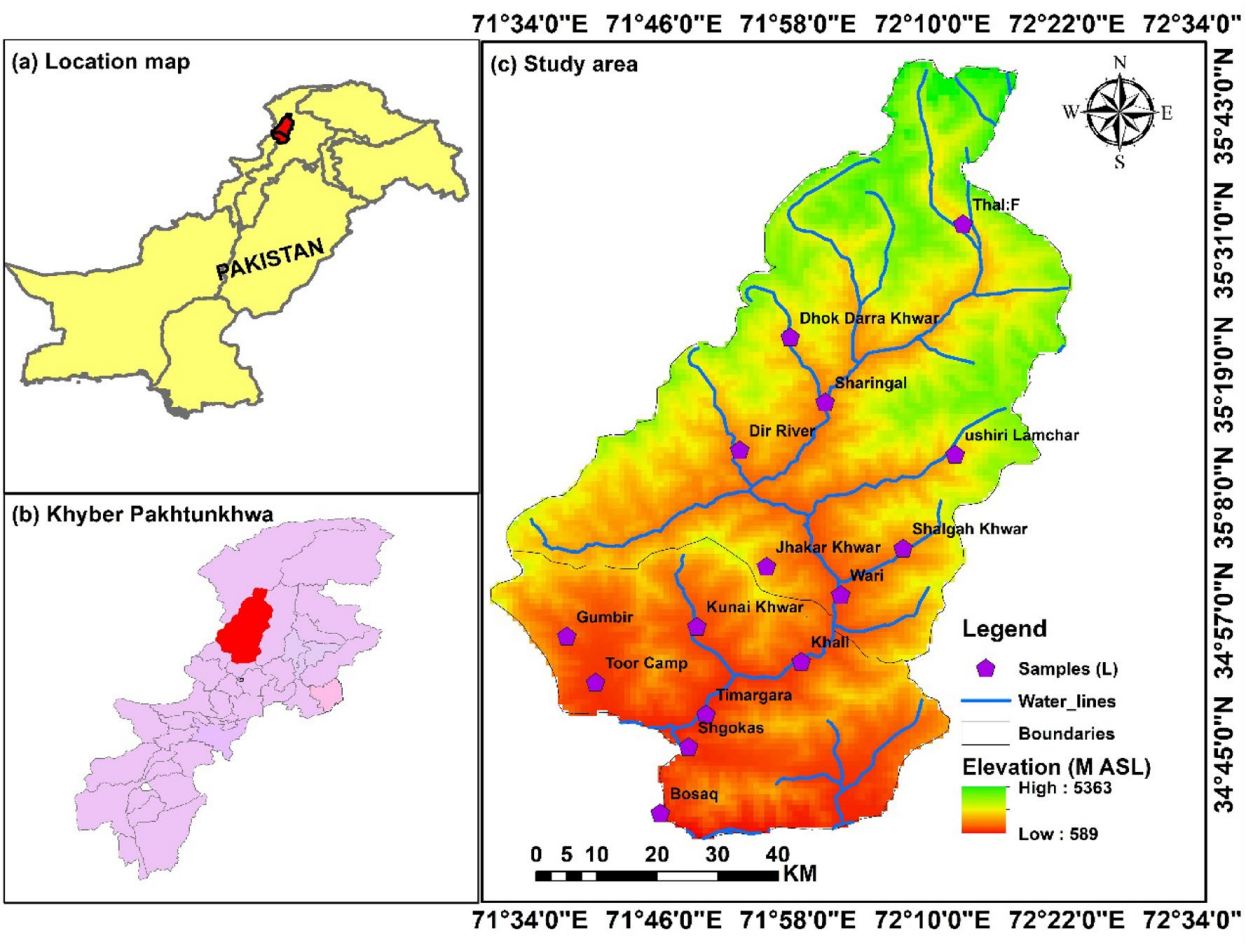
Shows the sampling collection site in the study area (Dir Upper and Lower). The software ArcGIS (version 10.5; https://malagis.com/arcgis-enterprise-105-download.html) was used to draw the map of the country, province, and detail study area.

The total population of 767,409, and most of the population is located on the bank of the river. Sewage, industrial, and agricultural wastewater are entering into rivers without treatment, these are the main sources of water pollution in the study area. Dir is split into the Upper Dir and Lower Dir districts (Fig. 1). The software ArcGIS (version 10.5) was used to draw the country, province, and detail study area discussed here. Dir's topography is dominated by mountains and hills that are a part of the Hindukush ranges. The mountain ranges run from north to south and from northeast to southwest along the northern borders with Chitral District. The Panjkora River enters the district from the northeast and flows southwest along the Bajour Agency's border until it joins the Swat River. Several streams in the Lower Dir and Dir River, the major stream of the Upper Dir, combine to form Panjkora River.

### Sampling techniques

The random sampling technique was used for the current study. The samples were taken from seven different locations in River Panjkora. Three samples were collected from the Upper Dir area (Wari, Sheringle, and Thal) and four samples were collected from the Dir Lower area (Khall, Timergara, Shagokas, and Bosaq) respectively. From each location, one fish and one water sample were collected for HM analysis. The rest of the samples were collected from the tributaries of river Punjkora. From each tributary one sample was collected and the following are the main tributaries of river Punjkora i.e. Ushiri Lamchar, Dhok Darra Khwar, Dir River, Shalgah Khwar, Jhakar Khwar, Kunai Khwar, Gumbir, Toor Camp respectively.

### Water sampling and analysis

Water samples were gathered in a polythene bottle. The bottles were clean, well-washed, and airtight. The water samples were collected from at middle position and the depth of water samples was 1 foot below the surface water. A total of fifteen samples were collected along the tributaries of river Panjkora. The samples were collected between March 2021 and April 2022. All samples were gathered in a 1-L bottle and preserved with 5 ml NHO_3_. Samples were sent to the "Advance Hydro-Geochemistry Laboratory, Department of Environmental Sciences, Quaid-I-Azam University Islamabad" for atomic absorption spectrophotometry. The samples were kept at 4 °C in the aforementioned laboratory and were examined within three months^44^.

A 250 volumetric flask was filled with 100 ml of water to remove the turbidity, and 5 ml of NHO_3_ (55%) was added to increase the sample's acidity. The acidified samples in the fume hood evaporated to a volume of 20 ml on a heated plate. After that, the samples were removed from the hot plate and left to cool at room temperature. The cooled samples received an additional 5 ml of NHO_3_, which was then again evaporated to produce 20 ml. After chilling at room temperature, the samples are diluted to 100 ml with tape water. Atomic absorption spectrophotometry (Spectra AA 2000) was employed to detect heavy elements like Zn, Pb, As, Cd, Fe, and Mg in the samples. To create the standard curves, characteristic standard solutions for each HMs were made and aspirated into a flame atomic absorption spectrophotometer (Spectra AA 2000). The curve is used to read the concentration of a particular HMs. To measure the concentration of HMs, ppm units were used.

### Fish sampling and analysis

The sample of fish was collected from the seven locations of the river Panjkora by a skilled fisherman and washed several times with distilled water. The sample was sealed in plastic bags with an ice box and transported to the laboratory for further analysis. In the laboratory, the fish sample was dissected with stainless scissors and forceps. The muscle of the fish was cut into small pieces and then dehydrated in the oven at 80 °C to obtain a constant weight. The dry sample converts into a fine powder with the help of mortar and pestle and is stored in desiccators until digestion. After this, 1 g of the sample was digested with 6 ml of 60% HNO_3_ and 3 ml of 35% H_2_O_2_ in a digestion flask for one hour. After that, the sample was cooled and filtered with Whatman filter paper 42. Finally, the filtrate obtained was diluted to 50 ml by adding deionized water in a volumetric flask and shifted for HMs analysis through an atomic absorption spectrophotometer. The analysis was performed in triplicate for quality assurance^45^.

### *Human health risk assessment of HMs *via* consumption of fish*

Different methods have been used for the determination of the risk associated with the consumption of contaminated fish. Estimated daily intake is one of the most common methods to help identify the number of pollutants consumed daily. For determining health risk related to HM via the consumption of fish were determined by using the following indices^46,47^.

### Estimated daily intake of HMs (EDI)

Daily intake of HMs via consumption of fish was determined by using the following equation:

1 $${\text{EDI}}=\frac{{\text{CM}}\times {\text{FIR}}}{{\text{WAB}}}$$

where EDI is the estimated daily intake of HMs, CM means the concentration of HMs in fish mg/kg wet weight, and FIR is the fish intake rate in the study area. It was considered 10 g/person/day because no information was available on daily fish intake and WAB representing the average body weight was taken 60 kg reported for fish from river Chenab, Pakistan^48^.

### Health risk index (HRI)

Health risk due to the eating of contaminated fish with metal was determined by using HRI. The value of HRI less than 1 is said to be safe for the exposed population. Where HRI above 1 means that there is a chance of non-carcinogenic effect on the exposed population. To determine HRI, the following equation was used^49^.

2 $${\text{HRI}}=\frac{{\text{EDI}}}{\mathrm{ RFD }}$$

where RFD is the Oral Reference Dose of the metal (mg/kg/day) and EDI is the estimated daily intake of HMs via fish consumption. *R*F*D* is an approximation of the daily tolerable exposure to which a person is expected to have without any adverse health effects during a lifetime. Values of RfD for Fe (0.7 mg kg^−1^per day), Pb (0.03 mg/kg/day), Mn (0.14 mg/kg/day), Zn (0.30 mg/kg/day), Cd (0.001 mg/kg/day), and As (0.0003 mg/kg/day) were taken from Integrated Risk Information System^50,51^.

### Target cancer risk (TCR)

The target cancer risk was calculated using the equation below. The carcinogenic risk of HMs is calculated using the target cancer^52^.

3 $$TCR=\frac{\mathrm{EF }\times {\text{ED}}\times {\text{FIR}}\times {\text{CM}}\times {\text{CPSo}}}{{\text{WAB}}\times {\text{ATc}}}$$

where TR is the target cancer risk, EF exposure frequency (365 days/year), ED exposure duration (70 years), FIR fish ingestion rate (10 g/person/day for this study CM concentration of HMs (mg/kg) WAB average body weight (60 kg) ATc is the averaging time, carcinogens (365 days/year for 70 years) used by USEPA^53^.While CPSo is the carcinogen potency slope factor which was determined by USEPA. While Mn, Fe, and Zn do not cause any carcinogenic effect, their CPSo has not yet been established by USEPA, so TR was only calculated for As, Cd, and Pb in order to determine carcinogenic risk. The CPSo values for Pb are 0.009, Cd 0.6, and for As 1.5. TR value is also equal to CPSo × EDI^54^.

### Spatial distribution of HMs

Spatial distributions of heavy metal i.e., Fe, Pb, Mn, Zn, Cd, and As were shown with the help of IDW software. The highest and lowest values were shown with the help of the map. The color shows the concentration of different parameters in the study area^55^.

### Statistical analysis

Different statistical methods are used, namely, variance and standard deviation. Data were analyzed by different software such as MS Word, Excel, Sigma Plot, and SPSS^56,57^. The ArcGIS (version 10.5) were used to draw country, province and study area detail maps.

## Results

### Assessment of HMs in water and fish

**Figure 2:**
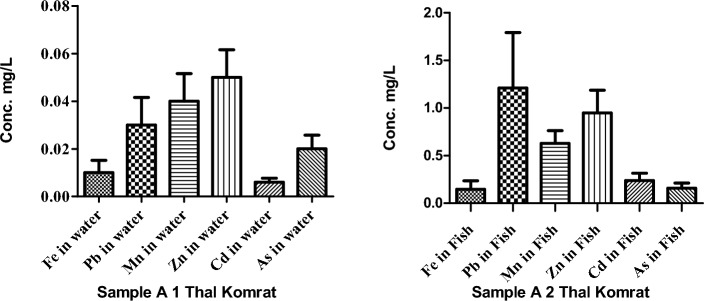
Concentrations of HMs in the water and fish of river Punjkora in Thal Komrat.

The concentration of heavy metal in water and fish in Thal Komrat valley are presented in Fig. 2. The concentration of Mn, 0.05 was detected in Thal Komrat valley (point A 1). Commonly, the concentration Pb was 1.21 detected in fish (point A 2).

**Figure 3:**
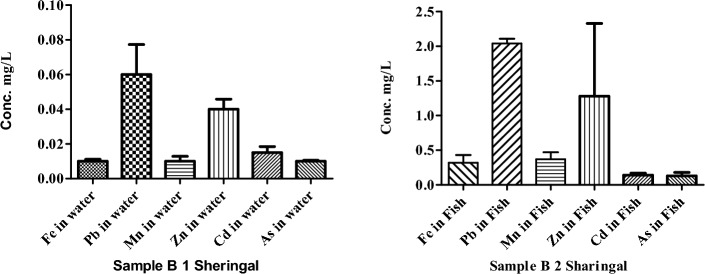
Concentrations of HMs in the water and fish of river Punjkora in Sharingal.

The concentration of HMs in water and fish samples of river Panjkora are presented in Fig. 3. The lowest values of HMs (Fe, Mn, and As) were found 0.01, 0.01, and 0.1 mg/L respectively in the water of Sharingal (point B 2). Similarly, at the location, Sheringal (point B 2), the concentrations of HMs (Pb, and Zn,) in fish samples were measured (2.04, 1.28, mg/L) respectively (point B 2).

**Figure 4:**
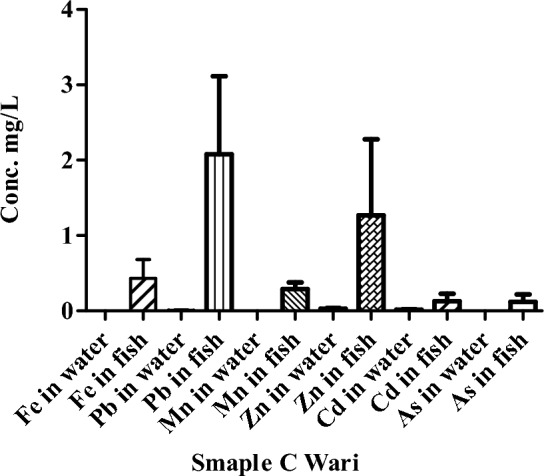
Concentrations of HMs in the water and fish of river Punjkora in Wari.

The concentration of HMs measured in the water of the river Punjkora at location Wari (point C) are presented in Fig. 4. The iron concentration was not detected at point C Wari. In the same way, the concentration of HMs (Fe, Pb, Mn, Zn, Cd, and As) was also measured in fish samples (0.43, 2.08, 0.29, 1.27, 0.13, 0.12 mg/kg) respectively at the location of Wari (point C).

**Figure 5:**
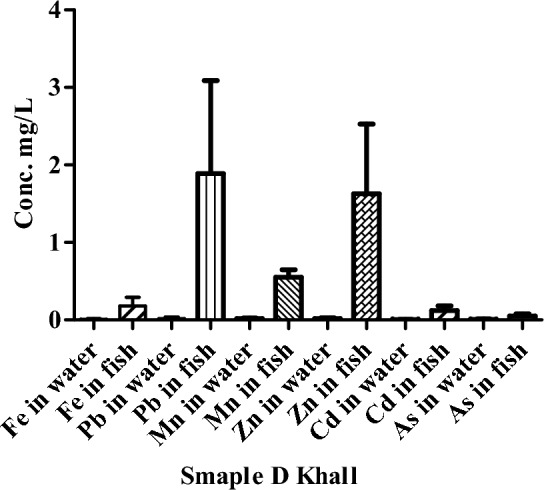
Concentration of HMs in the water of river Punjkora in Khall.

The concentration of HMs in water and fish samples of river Punjkora in Khall are presented as Fig. 5. The concentration of HMs was measured in the water of the river Punjkora. Similarly, the highest concentrations of Pb, were 1.89, mg/L in the fish at the location of Khall (point D).

**Figure 6:**
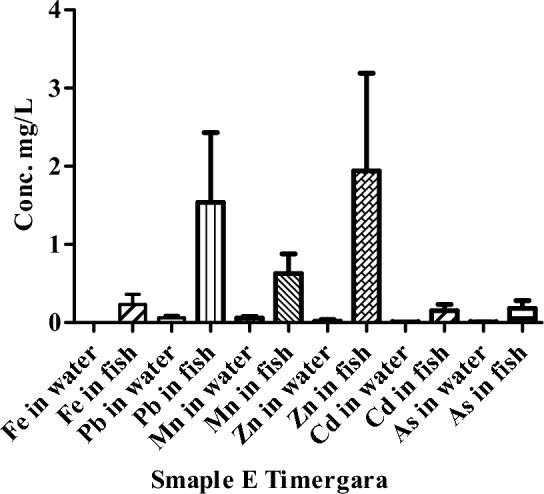
Concentration of HMs in the water of river Punjkora in Timergara.

The number of HMs in water and fish samples of river Punjkora at location Timergara is shown in Fig. 6. The lowest concentration of Cd was 0.008 mg/L in Timergara (point E). The concentrations of Fe and Pb were not detected at point E Timergara. The value of Mn was 1.94 mg/L was detected in the location of Timergara (point E).

**Figure 7:**
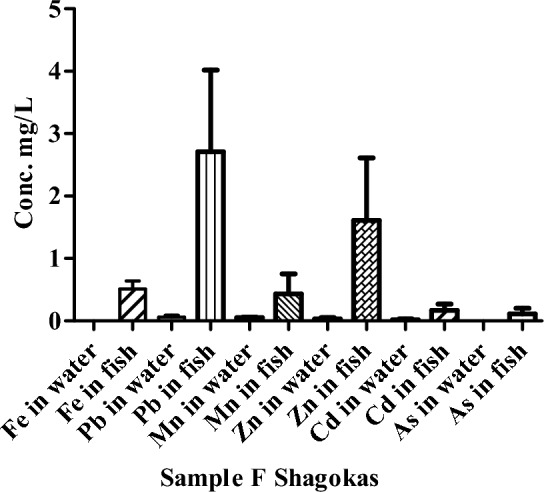
Concentrations of HMs in the water and fish of river Punjkora in Shagokas.

The concentration of HMs in the water and fish sample of river Punjkora in Shagokas (point F) is presented as Fig. 7. The concentrations for Fe and As were not detected at this point. Similarly, the concentrations of selected HMs were also measured in the fish samples of river Punjkora. The highest value of Pb were recorded in the fish at the location of Shagokas (point F).

**Figure 8:**
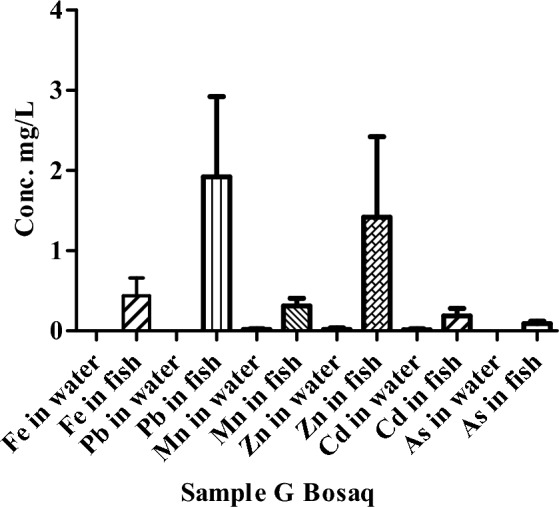
Concentrations of HMs in the water and fish of river Punjkora in Bosaq.

The number of HMs in the water and fish sample of river Panjkora in Bosaq (point G) is shown in Fig. 8. The concentrations of selected HMs were measured in the water of the river Punjkora. The concentrations of Fe, Pb, and As were not detected. Similarly, the concentrations of HMs, were also measured in fish of river Punjkora. The lowest value of As was recorded in the fish samples at the location of Bosaq (point G).

### Spatial distribution of HMs in tributaries and river Panjkora

**Figure 9:**
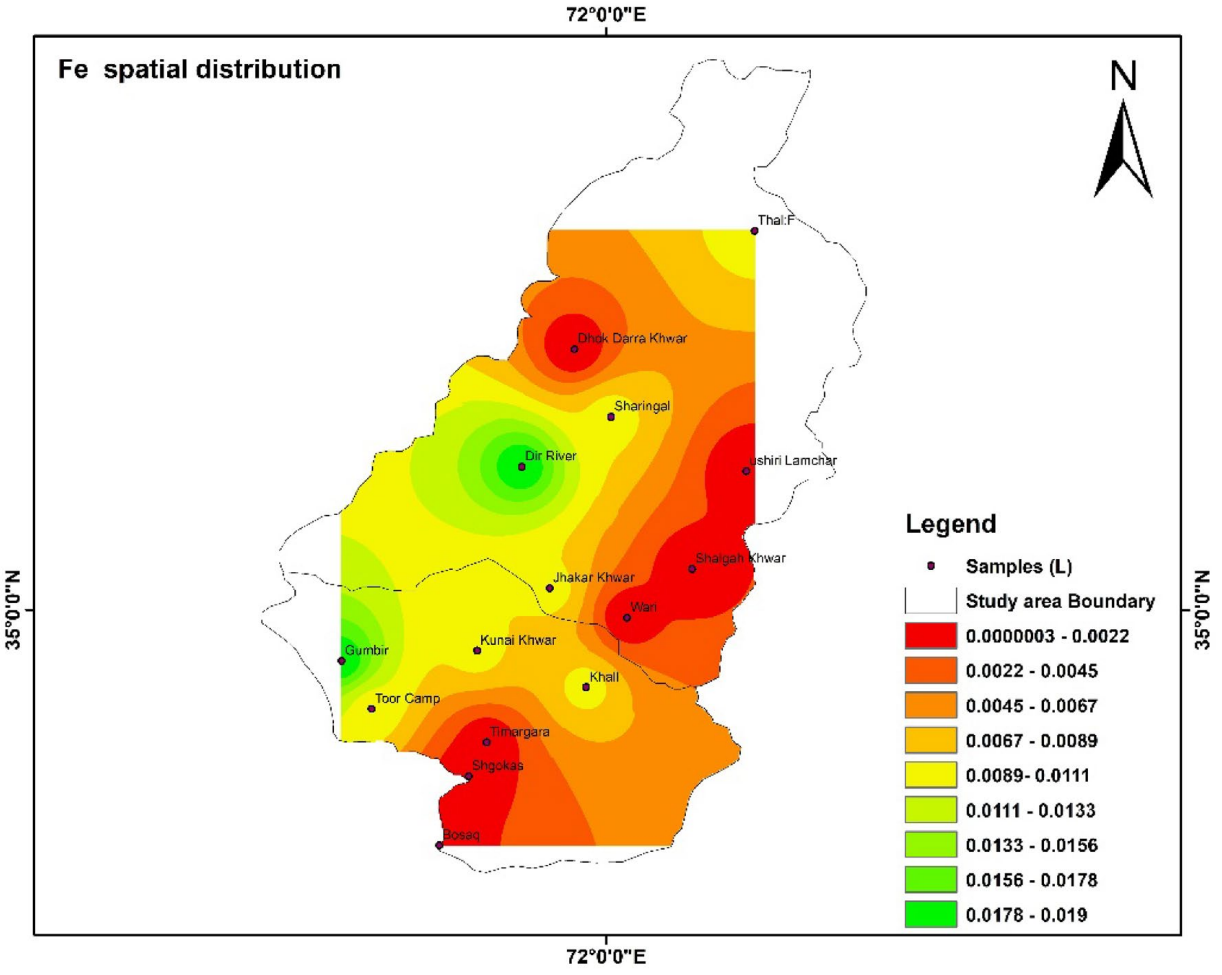
Show the spatial distribution of Fe in the study area. The software ArcGIS (version 10.5; https://malagis.com/arcgis-enterprise-105-download.html) was used to draw the map of the country, province, and detail study area.

**Figure 10:**
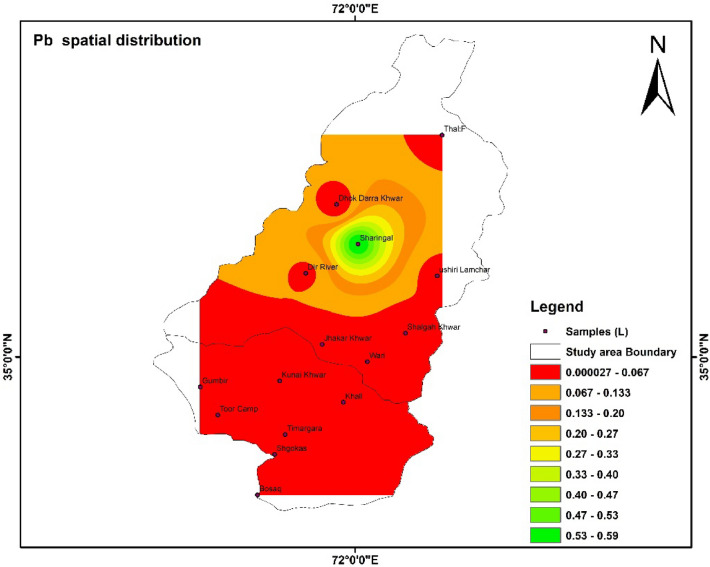
Show the spatial distribution of Pb in the study area. The software ArcGIS (version 10.5; https://malagis.com/arcgis-enterprise-105-download.html) was used to draw the map of the country, province, and detail study area.

The concentration Fe in the study area is presented in the Fig. 9. The concentrations of Fe were spatially distributed in the study area. The concentration of Fe in the tributaries of the river Panjkora river i.e., Dir River, Jhakar Khwar, Kunai Khwar, Gumbir, Toor Camp were shown with help of map While the concentration of Fe in Ushiri Lamchar, Dhok Darra Khwar, and Shalgah Khwar was not detected in water samples. Figure 10 shows the concentration of Pb in different tributaries of river Panjkora. The value of Pb was 0.05 mg/L and was detected in Dir River.

**Figure 11:**
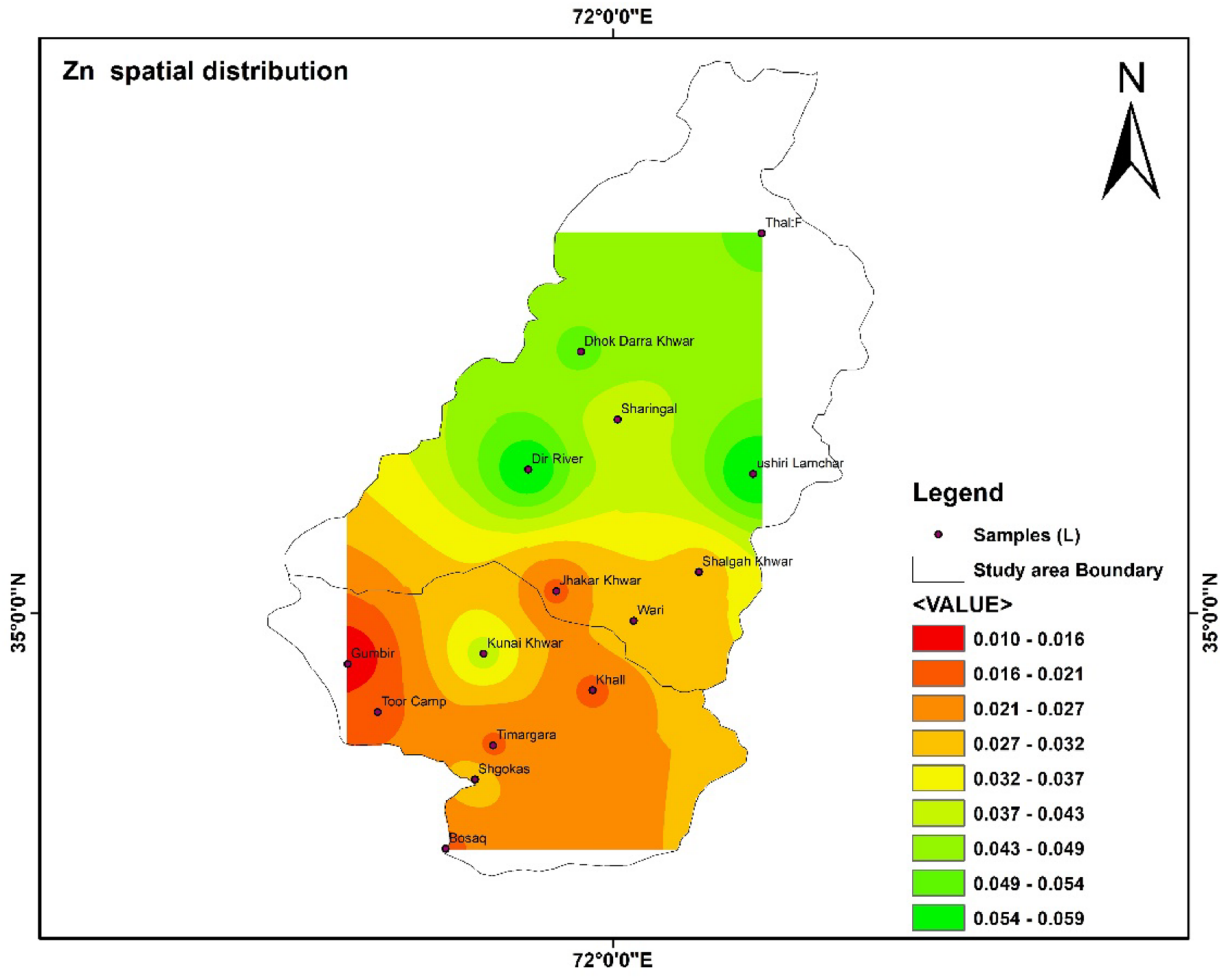
Show the spatial distribution of Zn in the study area. The software ArcGIS (version 10.5; https://malagis.com/arcgis-enterprise-105-download.html) was used to draw the map of the country, province, and detail study area.

**Figure 12:**
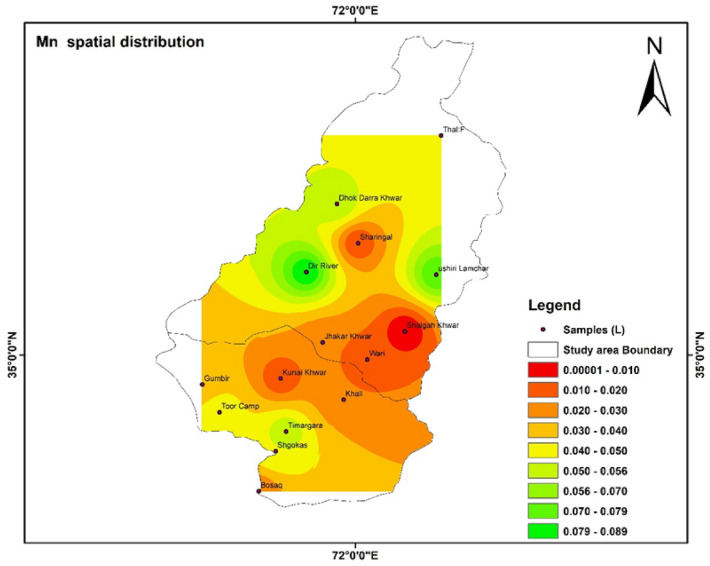
Show The spatial distribution of Mn in the study area. The software ArcGIS (version 10.5; https://malagis.com/arcgis-enterprise-105-download.html) was used to draw the map of the country, province, and detail study area.

In Fig. 11 the concentration Zn in the study area is presented. In the tributaries of river Panjkora the lowest vale of Zn was 0.01 detected in Gumbir. In Fig. 12 Mn concentration in water tributaries were spatially distributed in Upper and Lower Dir. The concentration of Mn in Shalgah Khwar was not detected in the study area.

**Figure 13:**
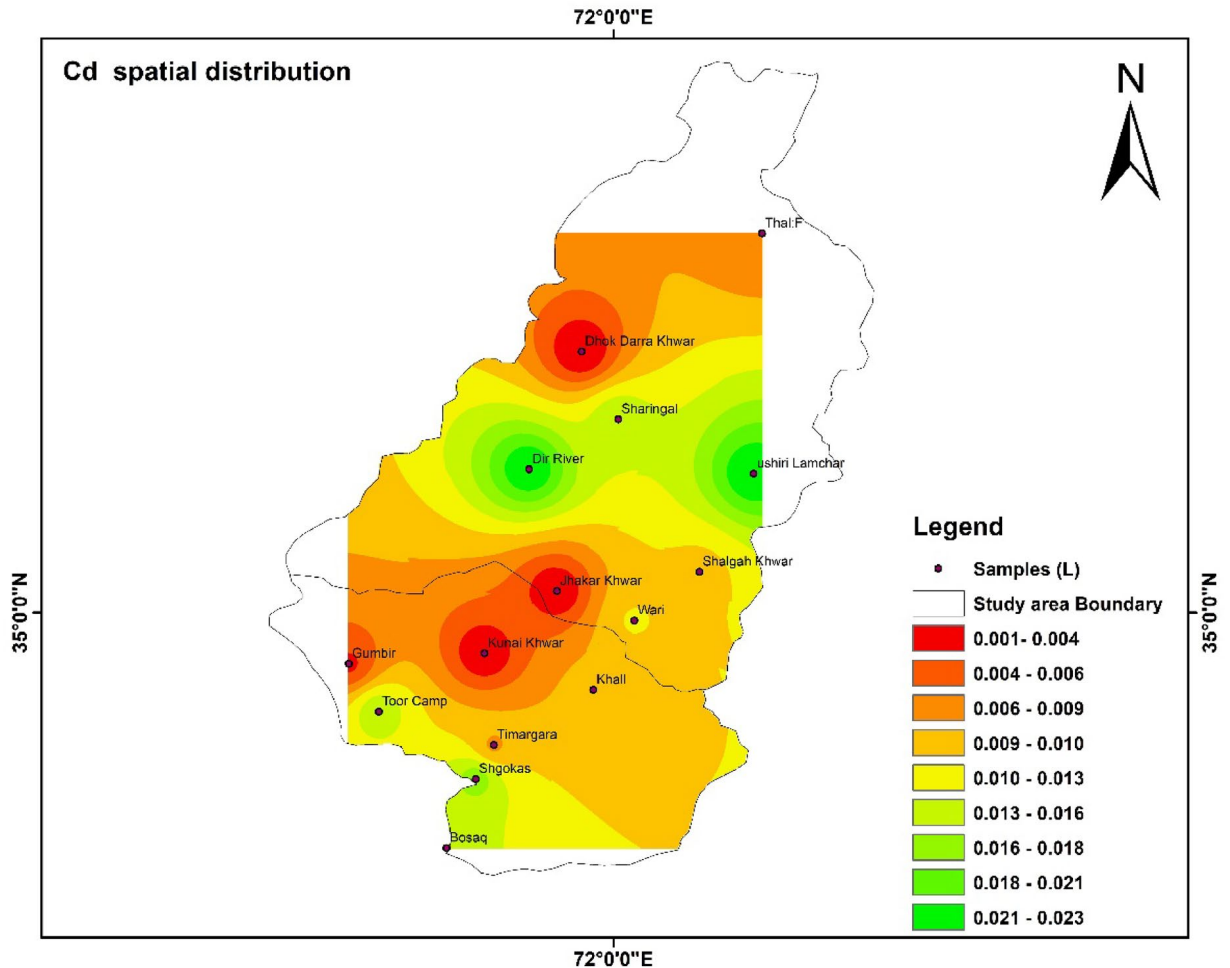
Show the spatial distribution of Cd in the study area. The software ArcGIS (version 10.5; https://malagis.com/arcgis-enterprise-105-download.html) was used to draw the map of the country, province, and detail study area.

**Figure 14:**
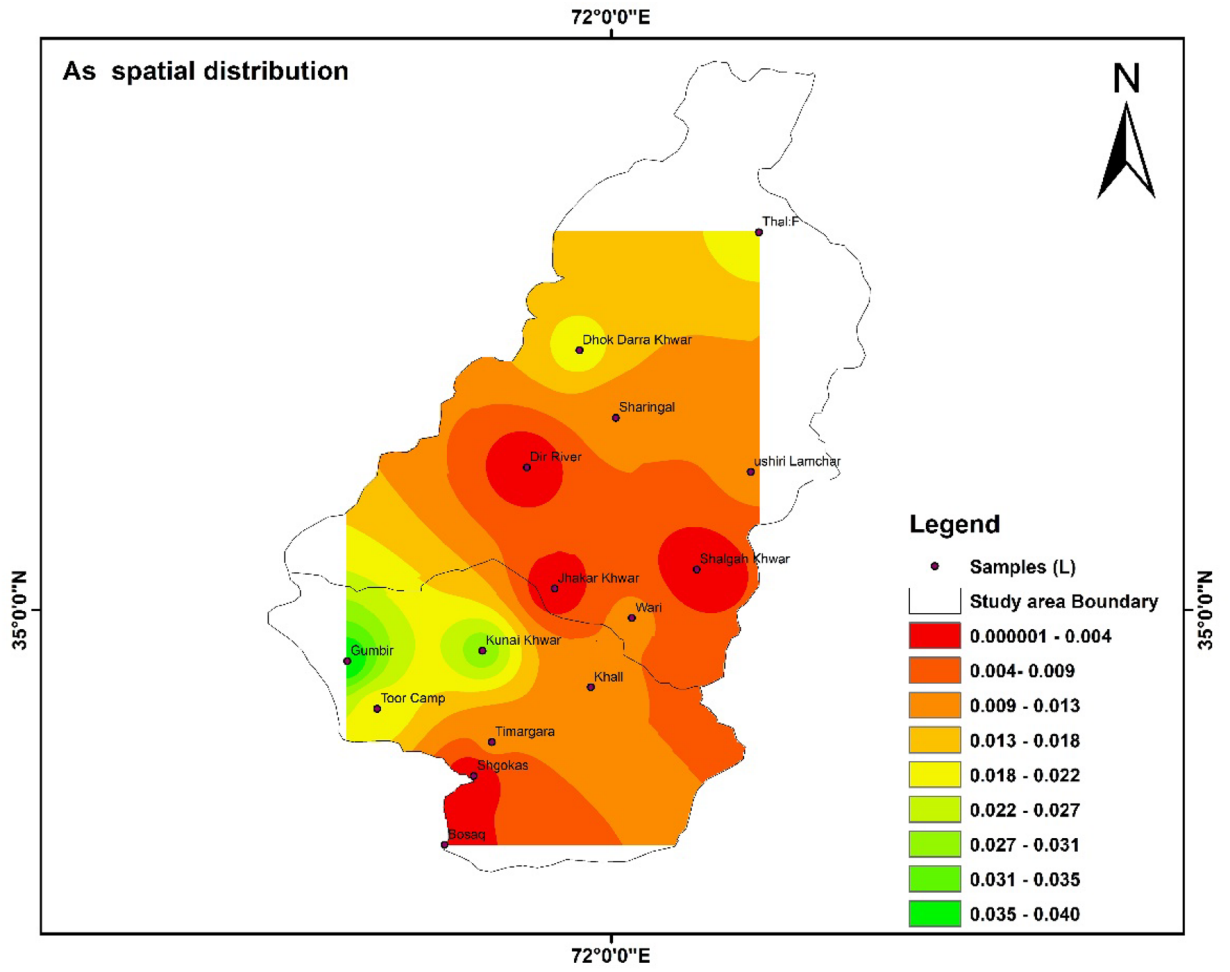
Shows the spatial distribution of As in the study area. The software ArcGIS (version 10.5; https://malagis.com/arcgis-enterprise-105-download.html) was used to draw the map of the country, province, and detail study area.

Figure 13 shows spatial distribution of heavy metal cadmium in different tributaries of river Panjkora. The concentration of Cd was distributed spatially. Figure 14 show spatial distribution of As concentration in the study area. Respectively, the concentration of As in Dir River, Shalgah Khwar, Jhakar Khwar, were not detected in these tributaries of river Punjkora.

### Health risk assessment

Risks to human health via consumption of HMs contaminated fish were determined by using health risk indices EDI and HRI by using Eqs. 1, 2, and 3, respectively and their results are given in Tables 1, 2, and 3.

**Table 1:** EDI of HM via consumption of contaminated fish.

Heavy metals	Sample locations						
TR	Point A	Point B	Point C	Point D	Point E	Point F	Point G
Fe	0.053	0.071	0.03	0.038	0.085	0.073	0.041
0.0561	N/A						
Pb	0.34	0.345	0.315	0.256	0.451	0.32	0.20
0.318	0.003042						
Mn	0.061	0.048	0.091	0.105	0.071	0.051	0.105
0.0764	N/A						
Zn	0.20	0.21	0.27	0.32	0.26	0.23	0.15
0.239	N/A						
Cd	0.023	0.021	0.02	0.025	0.028	0.031	0.04
0.027	0.015						
As	0.021	0.02	0.008	0.03	0.018	0.015	0.02
0.019	0.027

**Table 2:** HRI of HM and their mean via consumption of fish.

Heavy metals	Sample location							
	Mean	Point A	Point B	Point C	Point D	Point E	Point F	**Point G**
HRI location								
Fe	0.075	0.101	0.042	0.054	0.121	0.104	0.058	0.0792
Pb	11.33	11.5	10.50	8.33	15	10.66	6.66	10.56
Mn	0.43	0.342	0.65	0.75	0.507	0.364	0.75	0.541
Zn	0.7	0.70	0.9	1.06	0.86	0.76	0.5	0.782
Cd	23	21	20	25	28	31	40	26.85
As	70	66.66	27.66	100	60	50	62.99	66.66

**Table 3:** Target cancer risk (TCR) of HM in the study area.

HMs	EDI mg/kg /bw/day	CPSo	TCR
Fe	0.0561	N/A	N/A
Pb	0.318	0.009	0.002862
Mn	0.0764	N/A	N/A
Zn	0.239	N/A	N/A
Cd	0.027	0.6	0.0162
AS	0.019	1.5	0.0285

The results of the estimated daily intake of HMs via contaminated fish are given in Table 1. The maximum daily intake of Fe, Pb, Mn, Zn, Cd, As were occurring at 0.08, 0.451, 0.105, 0.32, 0.04, 0.03 mg/kg/day in points E, E, D, and G, D, G, D while the minimum daily intake occurs of 0.03, 0.20, 0.048, 0.15, 0.02 and 0.008 in point C, G, B, G, C, C. So, it was clear from the result that the maximum daily intake of HMs occurred for Pb and Zn. Similarly, the daily intake of HMs in all sample points of the river Panjkora follows the order of Pb > Zn > Mn > Fe > Cd > As. The EDI values for Pb, Cd, and As were found higher than their corresponding reference doses. While the rest HMs have lower EDI than the reference dose, so they do not pose non-carcinogenic health risks in the short term.

The health risk index (HRI) for individual HMs calculated from Eq. 2 and presented in the Table 2. The results showed that the mean HRI for Fe, Pb, Mn, Zn, Cd, and As were found 0.079 mg/kg/day, 10.56 mg/kg/day, 0.541 mg/kg/day, 0.782 mg/kg/day, 26.85 mg/kg/day and 62.91 mg/kg/day. HRI values for Fe ranged from (0.121–0.042) Pb (15–8.33) Mn (0.75–0.342) Zn (1.06–0.5) Cd (31–40) As (100.0–27.66). The highest mean HRI in all sample locations was recorded for As, Cd, and Pb. Overall, the HRI value of HMs decreased in the order of As > Cd > Pb > Zn > Mn > Fe. While the rest of the heavy metals Fe, Zn, and Mn have HRI < 1 while in point D, Zn has HRI greater than 1 so the exposed population is safe. The high HRI values of these metals are due to the high daily intake of HMs in the given area.

The results for TCR calculated from Eq. 3 are shown in Table 3. The target cancer risk was calculated only for As, Cd, and Pb because the CPSo value was only calculated for these metals while the other metals Fe, Zn, and Mn have no CPSo value so the TCR value was not calculated for the TCR value for Pb as found 0.002862 or 2.8 × ${10 }^{-3}$, for Cd its value was 0.016 or 1.6 $\times {10}^{-2}$ and for As the TCR was calculated 0.028 or 2.8 $\times {10}^{-2}$. The highest value was calculated for As 0.028 or 2.8 $\times {10}^{-2}$, while the lowest found for Pb was 0.002862 or 3.8 × ${10}^{-3}.$

## Discussion

Throughout the world, pollution of water with HMs is the main problem^58^. HMs is the most toxic source of water pollution in the river Punjkora. Most of the heavy metals in the water were above the permissible limits of that prescribed by WHO and Pak-EPA. The results show that the concentration of HMs in water such as Fe and As were detected less as compared to Pb, Mn, Zn, and Cd. The concentration of Fe was 0.01 mg/L in Sheringal Khall and Thall while in Wari, Bosaq, Shagokas and Timergara the concentration of Fe was not detected. The value of Fe was high in the tributaries of the river was 0.02 mg/L in Dir River and Gumber. The average concentration of Fe in upstream and downstream were 0.56 and 0.96 mg/L in the water of Tembi River^59^. The concentration of As was not detected in Bosaq and Shagokas and the concentration of As was 0.04 and 0.03 mg/L in Kunai Khwar and Gumbir tributaries. In Bangladesh Korotoa River, the concentration of As was 46 mg/L^60^.

Our study shows that the concentration of Pb were 0.06, 0.06, 0.05, 0.04, and 0.03 mg/L in Sheringal, Shagokas, Usheri Lamchar, Dir River, and Thall. In river Ganga, Lead is very toxic, and its concentration was higher in water (37–163 μg/L^61^. The values of Mn were 0.09, 0.08, 0.06, and 0.06 mg/L in Dir River, Ushiri Lamchar, Dhok Darra Khwar, Timergara. In river Ekiti the concentration of Mn were 0.12–0.30^62^. The concentration of Zn were 0.06, 0.06, 0.05, 0.05 were recorded in Ushiri Lamchar, Dir river, Dhok Darra Khwar, and Thall respectively, the lowest concentration was 0.01 mg/L in Gumbir. The concentration of Zn was 0.526 mg/L in water at at river Nile^63^. The concentration of Cd was 0.023 mg/L in Dir River, ushiri Lamchar, while the lowest vale was 0.001 mg/L in Dhok Darra Khwar, Jhakar Khwar, Kunai Khwar. In in Niger River water River the concentration of Cd was 50 mg/L in Nigeria’s^64^. The highest value of As was recorded in Gambir, while the concentrations in Shalgah Khwar, Jhakar Khwar, Shagokas, and Bosaq were not detected. The concentration of HMs was high in the tributaries and put pressure on river Panjkora.

The species of fish, i.e., Crossocheilusdiplocheilus, was selected due to the high consumption ratio within the study area. The concentrations of Fe, Pb, Mn, Zn, Cd, and As were also measured in fish because bioconcentration of HMs is taking place. The results show that the concentration of HMs was also measured in fish from the selected point of Panjkora river. The concentration of Fe was 0.51 and 0.44 mg/L in Shagokas and Bosaq. The lowest concentration of Fe was 0.18 mg/L in Khall. The ranged of Fe from 1.45–4.56 and 0.12–0.42 µg/g in fish (Districhodus rostratus) and (Heterotis niloticus) from river Benue in Nigeria^65^.

In Shagokas the highest value of Pb was 2.71 mg/L in Timergara. in the lowest value of Pb was 1.21 mg/L were detected in the Thall region. In different rivers, Pb concentrations were detected in the muscles of fish. The range of Pb was 0.337–0.810 mg/L in River Kabul^66^. Commonly, the highest value of Mn was 0.63 mg/L in Thall and Timergara. the lowest value of 0.29 mg/L was detected in Wari region. The concentration of Mn 34.98 ± 1.01, 22.42 ± 0.70, 29.09 ± 0.91, 125.81 ± 2.57, 27.89 ± 1.69 mg/kg ww Puntius ticto, Puntius sophore, Puntius chola, Labeo rohita, Glossogobius giuris from Buriganga river, Bangladesh^67^.

Respectively, the highest value of Zn 1.94 mg/L was detected in Timergara while the lowest value was 0.95 mg/L in Thall. The highest value of Cd was 0.24 mg/L in Thall region while the lowest value was 0.12 mg/L in Khall. The highest average values of Cd in fish (Schizothorax plagiostomus) were 1.4 ± 0.39 and 1.4 ± 0.44 µg g^−1^ in Swat Barandu River^68^The concentration of As was 0.18 mg/L was detected in Timergara while the lowest value was 0.05 mg/L in Khall. The value of As were 0.0091, 0.0025, 0.018, 0.0043, 0.0098 mg/Kg in Tor macrolepis, Glyptothorax stocki, Cyprinus carpio, Cirrhinus mrigala, Schizothorax plagiostomus in Swat river^69^.

The main source of HMs in the study area was anthropogenic activities such as mining, agricultural activities, municipal waste, domestic effluents, and industries. On another hand, there is no proper method used for the disposal of solid waste and all waste which was generated in the study area, enters into water bodies directly or indirectly. Rapid industrialization, urbanization, and agricultural activities are also the main sources of HMs. Due to continuous discharge, it pollutes the river with HMs^70^. On the other hand, rainwater is considered the main source of HMs because the study area is a sloping region. Rainwater is directly entering the river. Surface water is the main source of heavy metals that accumulate in fish^71^. The study area is the mountainous region, and the mountainous region has a rich source of metals and minerals. The mountain of Gwaldai area has 80% Pb, Bajaur Agency Has 35% Mn and Ushiri Valley has 16% Cu and mining activities are under processing which is the main cause of HMs. In the Swat region, surface water heavy metals such as Cd, Cr, Ni, and Pb demonstrated increasing pollution from upstream to downstream. This thread may be linked to the existence of fmafic and ultramafic rock formations, ongoing mining activities, as well as agricultural and industrial development downstream in the region of Sawat^72^.

The river Punjkora is considered the final disposal point of this waste. The waste (sewage and indusutrial wastewater) which was generated in Sharingal, Wari, Khall, and Timergara was disposed of on the bank of the river Punjkora. In Pakistan, agricultural runoff, and industrial and municipal waste have no proper way of disposal. Due to improper disposal HMs concentrations, i.e., Cu, Hg, Ni, Cd, As, Fe, Pb, and Zn were increasing day by day, which produced a bad impact on aquatic life and finally it will enter into the food chain. Commonly, this increases the level of HMs in water. Naturally, HMs entered into water bodies through rock due to their differences in chemical combinations. Besides these sources, the main sources of HMs in water were also in natural^40^.

### Risk assessment

Risk assessment for HMs via consumption of fish was determined by using parameters viz, EDI, HRI, and TCR. These risk assessment criteria were developed in the US by the EPA to determine the potential health risks posed by any chemical contaminants when exposed for an extended period of time^73^. The result of HRI from river Panjkora is shown in (Tables 1 and 2). In the study, a high daily intake of HMs was recorded for As, Cd, and Pb, which was found above their corresponding reference dose set by USEPA. Among these, more intakes occurred for Pb. While EDI for Fe, Zn, and Mn was found below their corresponding reference dose. A study conducted the daily intake of Pb (4.9 mg/kg bw/day) and Cd (0.30 mg/kg bw/day) in river Panjkora similarly they recorded the estimated daily intake (mg/kg bw/day) of Pb and Cd in river Swat and river Barandu as: 3.8, 0.23; 2.5, 0.23 respectively which was also found higher than the present study^29^. The estimated daily intake of Pb and As was recorded as 1.20 and 0.45 mg/kg/bw/day for fish in Cempaka Lake, Malaysia^74,75^. This value of Pb and As was also higher than in the present study.

In the present study, HRI values were found greater than 1 for As, Pb, and Cd. while for Mn, Fe, and Zn HRI was found less than 1 except Zn which has HRI > 1 in point D. HRI less than 1 means the exposed population has no noticeable adverse health effects, whereas HRI greater than 1 means that there is a risk of non-carcinogenic effects, with an increasing probability as the value increases^76,77^. The high HRI values result from the high EDI of the metals involved. The HRI value for Pb, As, and Cd in all sampling points is greater than 1, indicating a high risk of adverse health effects, especially for children and pregnant women from the consumption of fish products from this river.

Ekere, also, equally recorded HQ > 1 for some of the metals considered in this study in the rivers studied^78^. Studies conducted by Islam also revealed that HI values greater than 1 should produce public health distress for consumers of fishery yield^79,80^. From the results of the present study, HRI values were > 1 indicating potential health risks from consuming fish in river Panjkora The finding of this study is a source of distress due to potential health risk consequences from the intake of HMs via consumption of fish from river Panjkora.

The TCR calculated in this study showed that all metals were above the guideline value set by USEPA, which implies that fish consumption from river Panjkora is risky and may have the probability of cancer. The TR was categorized as TR ≤ 10^−6^ = Low; 10^−4^ to 10^−3^ = moderate; 10^−3^ to 10^−1^ = high and TR ≥ 10^−1^ = very high^78^. In this study, the TR value of Cd and As is high (${10}^{-2}$) while TR of Pb is moderate (${10}^{-3}$) which shows high cancer risk to the exposed population.

## Conclusions

In the study area, there is no proper sanitary system for the disposal of wastewater. On the other hand, all solid wastes which were generated in the study area were thronged to river water and its tributaries directly or indirectly. This waste produces an extra burden on the quality of water and increases the concentration of HMs in water. So, the present was conducted on HMs in water and fish in river Punjkora. Commonly, all of the HM’s concentrations were higher than normal, especially Pb concentrations were high so many times from normal because different anthropogenic activities were the main cause such as mining, industries, and car washing. Besides this, the concentrations of HMs in fish were high so many times in the study area. HRI assessment was carried out by using the health risk indices EDI, HRI, and TCR. Their results showed that fish intake from river Panjkora are not safe because the HRI value was found greater than 1. Similarly, carcinogenic health risk also showed a high risk of cancer. So, it was concluded from this study that fish products from river Panjkora is not safe for human consumption. Therefore, efforts should be made by the government to ensure the treatment of waste effluents from different sources before discharge into river Panjkora.

## Data Availability

All the data is available within the manuscript.

## References

[1] More TG, Rajput RA, Bandela NN (2003). Impact of heavy metals on DNA content in the whole body of freshwater bivalve, Lamelleidenmarginalis. Environ. Sci. Pollut. Res..

[2] Cüce H, Kalıpcı E, Ustaoğlu F, Kaynar İ, Baser V, Türkmen M (2022). Multivariate statistical methods and GIS-based evaluation of the health risk potential and water quality due to arsenic pollution in the Kızılırmak River. Int. J. Sedim. Res..

[3] Muhammad S, Usman QA (2022). Heavy metal contamination in water of Indus River and its tributaries, Northern Pakistan: Evaluation for potential risk and source apportionment. Toxin Rev..

[4] Stankovic S, Kalaba P, Stankovic AR (2014). Biota as toxic metal indicators. Environ. Chem. Lett..

[5] Islam MS, Islam ARMT, Phoungthong K, Ustaoğlu F, Tokatli C, Ahmed R, Ibrahim KA, Idris AM (2022). Potentially toxic elements in vegetable and rice species in Bangladesh and their exposure assessment. J. Food Compos. Anal..

[6] Mehmood, K., Ahmad, H. R., Abbas, R., Saifullah, & Murtaza, G. Heavy metals in urban and peri-urban soils of a heavily-populated and industrialized city: Assessment of ecological risks and human health repercussions. *Hum*. *Ecol*. *Risk**Assess*. *Int*. *J*. **26**(6), 1705–1722. 10.1080/10807039.2019.1601004 (2019).

[7] Nawab J, Khan N, Ahmed R, Khan S, Ghani J, Rahman Z, Khan F, Wang X, Muhammad J, Sher H (2019). Influence of different organic geo-sorbents on *Spinacia*
*oleracea* grown in chromite mine-degraded soil: A greenhouse study. J. Soil Sediment.

[8] Kalipci E, Cüce H, Ustaoğlu F, Dereli MA, Türkmen M (2023). Toxicological health risk analysis of hazardous trace elements accumulation in the edible fish species of the Black Sea in Türkiye using multivariate statistical and spatial assessment. Environ. Toxicol. Pharmacol..

[9] Florea AM, Büsselberg D (2006). Occurrence, use and potential toxic effects of metals and metal compounds. Biometals.

[10] Ustaoğlu F, Tepe Y, Aydın H, Akbaş A (2017). Investigation of water quality and pollution level of lower Melet River, Ordu, Turkey. Alinteri J. Agric. Sci..

[11] Ahmad K, Muhammad S, Ali W, Jadoon IAK, Rasool A (2020). Occurrence, source identification and potential risk evaluation of heavy metals in sediments of the Hunza River and its tributaries. Gilgit-Baltistan. Environ. Technol. Innov..

[12] Dawson E, Macklin M (1998). Speciation of heavy metals in floodplain and flood sediments: A reconnaissance survey of the aire valley, west Yorkshire, Great Britain. Environ. Geochem. Health.

[13] Gupta N, Khan DK, Santra SC (2008). An assessment of heavy metal contamination in vegetables grown in wastewater-irrigated areas of Titagarh, West Bengal, India. Bull. Environ. Contam. Toxicol..

[14] Lan T, Hu Y, Cheng L, Chen L, Guan X, Yang Y, Guo Y, Pan J (2022). Floods and diarrheal morbidity: Evidence on the relationship, effect modifiers, and attributable risk from Sichuan Province, China. J. Glob. Health.

[15] Chaoua S, Boussaa S, El Gharmali A, Boumezzough A (2019). Impact of irrigation with wastewater on accumulation of heavy metals in soil and crops in the region of Marrakech in Morocco. J. Saudi Soc. Agric. Sci..

[16] Bai B, Chen J, Bai F, Nie Q, Jia X (2024). Corrosion effect of acid/alkali on cementitious red mud-fly ash materials containing heavy metal residues. Environ. Technol. Innov..

[17] Ustaoğlu F, Taş B, Tepe Y, Topaldemir H (2021). Comprehensive assessment of water quality and associated health risk by using physicochemical quality indices and multivariate analysis in Terme River, Turkey. Environ. Sci. Pollut. Res..

[18] Bai B, Nie Q, Zhang Y, Wang X, Hu W (2021). Cotransport of heavy metals and SiO_2_ particles at different temperatures by seepage. J. Hydrol..

[19] Bashir N, Farid M, Saeed R, Tauqeer HM, Ali S, Rizwan M, Sallah-Ud-Din R (2017). Measurement of different heavy metals concentration in roadside dust in the vicinity of Gujrat, Pakistan. J. Sci. Chem..

[20] Xue Y, Ma Y, Long G, He H, Li Z, Yan Z, Wan J, Zhang S, Zhu B (2023). Evaluation of water quality pollution and analysis of vertical distribution characteristics of typical Rivers in the Pearl River Delta, South China. J. Sea Res..

[21] Azizullah A, Khattak MNK, Richter P, Hader DP (2011). Water pollution in Pakistan and its impact on public health. A review. Environ. Int..

[22] Le CV, Jensen JR (2014). Individual lift irrigation: A case study in the Cau Son irrigation and drainage area, Red River Basin, Vietnam. Paddy Water Environ..

[23] Shammout MW, Shatanawi K, Zamil MAL, Abualhaija MM (2023). a long-term estimation and modeling of domestic water resources in the Yarmouk River Basin-Jordan. Water Conserv. Manag..

[24] Otchere FAA (2019). 50-year review on heavy metal pollution in the environment: Bivalves as bio-monitors. Afr. J. Environ. Sci. Technol..

[25] Rogowska J, Cieszynska-Semenowicz M, Ratajczyk W, Wolska L (2020). Micropollutants in treated wastewater. Ambio.

[26] Ali H, Khan E, Ilahi I (2019). Environmental chemistry and ecotoxicology of hazardous heavy metals: Environmental persistence, toxicity, and bioaccumulation. J. Chem..

[27] Kanamarlapudi SLRK, Chintalpudi VK, Muddada S (2018). Application of biosorption for removal of heavy metals from wastewater. Biosorption.

[28] Malik RN, Hashmi MZ, Huma Y (2014). Trace metal accumulation in edible fish species from Rawal Lake Reservoir, Pakistan. Environ. Sci. Pollut. Res..

[29] Töre Y, Ustaoğlu F, Tepe Y, Kalipci E (2021). Levels of toxic metals in edible fish species of the Tigris River (Turkey); threat to public health. Ecol. Indic..

[30] Shahid M, Niazi NK, Ducat C, Naidu R, Khalid S, Rahman MM, Bibi I (2018). A meta-analysis of the distribution, sources and health risks of arsenic-contaminated groundwater in Pakistan. Environ. Pollut..

[31] Puttaiah, E.T., Kiran, B.R. Heavy metals transport in a sewage fed lake of Karanataka, India. In: The 12th World Lake Conference, 347–354. 10.25177/JESES.4.2.RA.482 (2008).

[32] Sattar AA (2021). Preparation of novel hybrid (almond shell and pleurotus sajor caju) biosorbent for the removal of heavy metals (nickel and lead) from wastewater. Water Conserv. Manag..

[33] Tokatlı C, Varol M, Ustaoğlu F (2023). Ecological and health risk assessment and quantitative source apportionment of dissolved metals in ponds used for drinking and irrigation purposes. Environ. Sci. Pollut. Res..

[34] Dukes AD, Eklund RT, Morgan ZD, Layland RC (2020). Heavy metal concentration in the water and sediment of the Lake Greenwood Watershed. Water Air Soil Pollut..

[35] Topaldemir H, Taş B, Yüksel B, Ustaoğlu F (2023). Potentially hazardous elements in sediments and Ceratophyllum demersum: An ecotoxicological risk assessment in Miliç Wetland, Samsun, Türkiye. Environ. Sci. Pollut. Res..

[36] Bai J, Huang L, Gao H, Zhang G (2017). Wetland biogeochemistry and ecological risk assessment. Phys. Chem. Earth.

[37] Shakir SK, Azizullah A, Murad W, Daud MK, Nabeela F, Rahman H, ur Rehman S, Häder DP (2017). Toxic metal pollution in Pakistan and its possible risks to public health. Rev. Environ. Contam. Toxicol..

[38] Hussain M, Muhammad S, Malik RN, Khan MU, Farooq U (2014). Status of heavy metal residues in fish species of Pakistan. Rev Environ Contam Toxico. Cumul. Compr. Index Subj. Cover..

[39] Sweet LI, Zelikoff JT (2001). Toxicology and immunotoxicology of mercury: A comparative review in fish and humans. J. Toxicol. Environ. Health Part B Crit. Rev..

[40] Rahman MM, Asaduzzaman M, Naidu R (2013). Consumption of arsenic and other elements from vegetables and drinking water from an arsenic-contaminated area of Bangladesh. J. Hazard. Mater..

[41] Ikem A, Egiebor NO, Nyavor K (2003). Trace elements in water, fish and sediment from Tuskegee Lake, Southeastern USA. Water Air Soil Pollut..

[42] Ullah S, Ahmad T (2015). Distribution of ABO and Rh (D) blood groups in the population of District Dir Lower, Khyber Pakhtunkhwa Pakistan. World Appl. Sci. J..

[43] Ali H, Khan E (2019). Bioaccumulation of Cr, Ni, Cd and Pb in the economically important freshwater fish *Schizothorax*
*plagiostomus* from three rivers of Malakand Division, Pakistan: Risk assessment for human health. Bull. Environ. Contam. Toxicol..

[44] Javed M, Usmani N (2016). Accumulation of heavy metals and human health risk assessment via the consumption of fresh water fish Mastacembelus armatusinhabiting, Thermal power plant effluent loaded canal. Springer Plus.

[45] Varol M, Sünbül MR (2018). Multiple approaches to assess human health risks from carcinogenic and non-carcinogenic metals via consumption of five fish species from a large reservoir in Turkey. Sci. Total Environ..

[46] Yi Y, Tang C, Yi T, Yang Z, Zhang S (2017). Health risk assessment of heavy metals in fish and accumulation patterns in food web in the upper Yangtze River, China. Ecotoxicol. Environ. Saf..

[47] Alamdar A, Eqani SAMAS, Hanif N, Ali SM, Fasola M, Bokhari H, Shen H (2017). Human exposure to trace metals and arsenic via consumption of fish from river Chenab, Pakistan and associated health risks. Chemosphere.

[48] Rawan B, Ullah W, Ullah R, Akbar TA, Ayaz Z, Javed MF, Khan O (2022). Assessments of roof-harvested rainwater in Disctrict Dir Lower. Khyber Pakhtunkhwa Pakistan. Water.

[49] USEPA. United States Environmental Protection Agency, Risk-based concentration table. Philadelphia. (Accessed 15 February 2022); https://semspub.epa.gov/work/05/229825 (2009).

[50] Wang X, Sato T, Xing B, Tao S (2005). Health risks of heavy metals to the general public in Tianjin, China via consumption of vegetables and fish. Sci. Total Environ..

[51] USEP. USEPA Regional Screening Level (RSL) Summary Table: November 2011. Available at: http://www.epa.gov/regshwmd/risk/human/Index.htm, last update: 20th January, 2014. (Accessed 15 March 2023) https://www.epa.gov/risk/regional-screening-levels-rsls-generic-tables (2011).

[52] USEPA. EPA Region III Risk-Based Concentration (RBC) Table 2008 Region III, 1650 Arch Street, Philadelphia, Pennsylvania 19103. (Accessed 25 May 2022) https://semspub.epa.gov/work/05/229825 (2012).

[53] USEPA. United States Environmental Protection Agency, Risk-based concentration table. Philadelphia (Accessed 17 Jun 2023) https://archive.epa.gov/region9/superfund/web/html/index-23.html (2009).

[54] Bhat NA, Ghosh P, Ahmed W, Naaz F, Darshinee AP (2023). Heavy metal contamination in soils and stream water in Tungabhadra basin, Karnataka: Environmental and health risk assessment. Int. J. Environ. Sci. Technol..

[55] Hassan N, Zhong Z, Wang D, Zhu Y, Naeen I, Ahungu AB, Wan HY, Li X (2023). Effects of long-term mowing on species diversity, biomass and composition of plant community in a semi-arid grassland in Northeastern China. Appl. Veg. Sci..

[56] Bilal, Q. W., Noman, M., Shen, Z., & Ali, K. S. Faisal khan, Kashif Ali Panhwar, Hamidova Emiliya, Ra-bia Tasleem, Javed Ahmad, Izhar Ul Haq, Muhammad Subhanullah, Zakir Ullah. Phytoremediation of contaminated soil Lead and Cadmi-um by *Brassica**júncea* (L.) Czern plant. *J*. *Earth**Sci*. *Environ*. *Stud*. **5**(4), 110–120 10.25177/JESES.5.4.RA.10693 (2020).

[57] Favorito R, Chiarelli G, Grimaldi MC, De Bonis S, Lancieri M, Ferrandino I (2011). Bioaccumulation of cadmium and its cytotoxic effect on zebrafish brain. Chem. Ecol..

[58] Shanbehzadeh S, Vahid Dastjerdi M, Hassanzadeh A, Kiyanizadeh T (2014). Heavy metals in water and sediment: A case study of Tembi River. J. Environ. Public Health.

[59] Islam MS, Ahmed MK, Raknuzzaman M, Habibullah-Al-Mamun M, Islam MK (2015). Heavy metal pollution in surface water and sediment: A preliminary assessment of an urban river in a developing country. Ecol. Indicat..

[60] Gupta V, Kumar D, Dwivedi A, Vishwakarma U, Malik DS, Paroha S, Mohan N, Gupta N (2023). Heavy metal contamination in river water, sediment, groundwater and human blood, from Kanpur, Uttar Pradesh, India. Environ. Geochem. Health.

[61] Samuel AO (2013). Level of selected metals in water, sediment and fish samples from Itapaji Dam. South-Western, Nigeria. Am. Chem. Sci. J..

[62] Ghannam HE (2021). Risk assessment of pollution with heavy metals in water and fish from River Nile, Egypt. Appl. Water Sci..

[63] Olatunji OS, Osibanjo O (2013). Eco-partitioning and indices of heavy metal accumulation in sediment and Tilapia zillii fish in water catchment of River Niger at Ajaokuta, North Central Nigeria. Int. J. Phys. Sci..

[64] Akan JC, Salwa M, Yikala BS, Chellube ZM (2012). Study on the distribution of heavy metals in different tissues of fishes from River Benue in Vinikilang, Adamawa State, Nigeria. Br. J. Appl. Sci. Technol..

[65] Ali H, Khan E (2018). Assessment of potentially toxic heavy metals and health risk in water, sediments, and different fish species of River Kabul, Pakistan. Hum. Ecol. Risk Assess. Int. J..

[66] Kawser Ahmed M, Baki MA, Kundu GK, Islam S, Islam M, Hossain M (2016). Human health risks from heavy metals in fish of Buriganga river, Bangladesh. SpringerPlus.

[67] Ali H, Khan E (2019). Bioaccumulation of Cr, Ni, Cd and Pb in the economically important freshwater fish Schizothorax plagiostomus from three rivers of Malakand Division, Pakistan: Risk assessment for human health. Bull. Environ. Contam. Toxicol..

[68] Liu M, Xu Y, Nawab J, Rahman Z, Khan S, Idress M, Ali A (2020). Contamination features, geo-accumulation, enrichments and human health risks of toxic heavy metal (loids) from fish consumption collected along Swat river, Pakistan. Environ. Technol. Innov..

[69] Muhammad S, Ullah S, Ali W, Jadoon IA, Arif M (2022). Spatial distribution of heavy metal and risk indices of water and sediments in the Kunhar River and its tributaries. Geocarto Int..

[70] Singh AK, Srivastava SC, Verma P, Ansari A, Verma A (2014). Hazard assessment of metals in invasive fish species of the Yamuna River, India in relation to bioaccumulation factor and exposure concentration for human health implications. Environ. Monit. Assess..

[71] Khan K, Lu Y, Khan H, Zakir S, Khan S, Khan AA, Wang T (2013). Health risks associated with heavy metals in the drinking water of Swat, Northern Pakistan. J. Environ. Sci..

[72] USEPA. Office of Water Regulations and Standard: United States Environmental Protection Agency, Washington, DC. (Accessed 09 February 2023). (1989).

[73] Taweel A, Shuhaimi-Othman M, Ahmad AK (2013). Evaluation of copper, lead and arsenic level in tilapia fish in Cempaka Lake (Bangi, Malaysia) and human daily/weekly intake. Biologia.

[74] Saha N, Zaman MR (2013). Evaluation of possible health risks of heavy metals by consumption of foodstuffs available in the central market of Rajshahi City, Bangladesh. Environ. Monit. Assess..

[75] Shang Y, Song K, Lai F, Lyu L, Liu G, Fang C, Hou J, Qiang S, Yu X, Wen Z (2023). Remote sensing of fluorescent humification levels and its potential environmental linkages in lakes across China. Water Res..

[76] Qin Y, Huang C, Huang G, Li H, Shohag MJI, Gu M, Shen F, Lu D, Zhang M, Wei Y (2023). Relative bioavailability of selenium in rice using a rat model and its application to human health risk assessment. Environ. Pollut..

[77] Ekere Nwachukwu R, Ihedioha Janefrances N, Eze Ifeanyi S, Agbazue Vitus E (2014). Health risk assessment in relation to heavy metals in water sources in rural regions of South East Nigeria. Inter. J. Phys. Sci..

[78] Islam MS, Ahmed MK, Habibullah-Al-Mamun M, Islam KN, Ibrahim M, Masunaga S (2014). Arsenic and lead in foods: A potential threat to human health in Bangladesh. Food Addit. Contam. Part A.

[79] NYSDOH, (New York State Department of Health). Hopewell precision area contamination: appendix C-NYS DOH. Procedure for evaluating potential health risks for contaminants of concern (Accessed 15 February 2022) http://www.health.ny.gov/environmental/investigations/hopewell/appendc.htm (2007).

[80] Zhu G, Liu Y, Shi P, Jia W, Zhou J, Liu Y, Ma X, Pan H, Zhang Y, Zhang Z, Sun Z, Yong L, Zhao K (2022). Stable water isotope monitoring network of different water bodies in Shiyang River basin, a typical arid river in China. Earth Sys. Sci. Data.

